# In Vitro Bioaccessibility of Bioactive Compounds of Freeze-Dried Orange Juice Co-Product Formulated with Gum Arabic and Modified Starch

**DOI:** 10.3390/molecules28020810

**Published:** 2023-01-13

**Authors:** Eva García-Martínez, María del Mar Camacho, Nuria Martínez-Navarrete

**Affiliations:** Food Investigation and Innovation Group, Food Technology Department, Universitat Politècnica de València, Camino de Vera s/n, 46022 Valencia, Spain

**Keywords:** in vitro digestion, co-product, antioxidant capacity, vitamin C, total phenols, carotenoids, citrus industry

## Abstract

The large amount of waste generated by the orange juice industry has sparked the interest of many researchers in incorporating recycling systems and following a much more sustainable circular economy model. This work proposes the valorization of the co-product generated in the orange juice extraction industry after freeze-drying for its subsequent reuse as a natural ingredient in the food industry. In addition, the possible protective effect of gum Arabic and corn starch esterified with octenyl succinic groups, in proportions optimised in previous studies 0.25 and 0.45 g/g orange co-product dry solutes, on the main bioactive compounds of orange peel during the freeze-drying process has been studied. The samples were characterised for their content of vitamin C (ascorbic and dehydroascorbic acids), flavonoids (hesperidin and narirutin), total phenols and total carotenoids, as well as their antioxidant capacity (DPPH and FRAP assays). In addition, samples were digested, mimicking the human enzymatic oral gastro-intestinal digestion process, and the bioaccessibility of the bioactive compounds was evaluated. It was observed that the addition of both biopolymers improved the stability of the hydrophilic compounds during freeze-drying. This conservative effect was more remarkable for higher biopolymer concentrations. However, no protective effect on carotenoid compounds was observed. This trend was reflected in the antioxidant activity of the different samples. In addition, the incorporation of biopolymers improved the bioaccessibility of the bioactive compounds studied. In conclusion, the results supported the feasibility of the freeze-dried orange juice co-product as a natural, sustainable source of health-promoting compounds.

## 1. Introduction

World production of oranges has been growing exponentially in recent years. In 2021, 48.8 million tonnes of oranges were produced worldwide, of which around 30 million tonnes were destined for direct consumption and 18 million tonnes for the production of preserves, jellies, juices, nectars, and so forth. The orange juice industry stands out within this group of processed products, with 1.7 million tonnes of juice in 2021 [1]. In the industrial juice process, only approximately 45% of the total weight of the fruit is used, while the rest, such as the peel (flavedo and albedo; 27%), the pulp (endocarp; 26%) and the seeds (2%), constitute the main by-product. Considering these numbers, it could be estimated that global orange juice production could generate between 0.8 and 1 million tonnes of by-products each year [2]. An alternative must be found to make this product loss profitable in line with the circular economy principle [3]. This sustainable conversion of agri-food waste into a value-added product supports the concepts of “zero waste” and “waste to wealth” [4]. Although much of this waste is destined for the animal feed sector, the biomass generated in the orange juice industry is rich in water, dietary fibre, proteins, minerals and other bioactive compounds (vitamins, phenolic compounds and carotenoids) with health benefits [5]. As described by Domínguez [6], the major components of dry citrus peel residues are dietary fibre (cellulose up to 35%, pectin up to 23% and hemicellulose up to 11% and lignin up to 8%) and sugars. Currently, the citrus processing industry has implemented processes to recover waste to extract health-promoting dietary fibres [4].

Nevertheless, the citrus peel also contains other components which, although minor, appear to contribute to a healthy diet. This is the case of vitamin C, polyphenols and carotenoids with provitamin A activity [7]. It is also rich in flavonoids, mainly hesperidin, neoshesperidin, naringin, narirutin, tangeretin and nobiletin [8]. All these compounds have shown numerous beneficial functions for human health, such as potent antioxidants and free radical scavengers, being considered agents that exert anticarcinogenic and cardioprotective action in our body. Moreover, dietary fibre and phenolic compounds have been shown to stimulate the growth of beneficial microorganisms, especially lactic acid bacteria and other probiotics. Thus, bioactive compounds present in the waste of the citrus industry emerge as potential prebiotic ingredients [9]. These compounds could also have applications in food stabilisation because they help inhibit oxidation (lipid and protein) and the growth of pathogenic and deteriorating bacteria in the food supply [10]. Therefore, investigating the possibility of reintroducing it into the human food chain would help add value to it and improve this industry’s efficiency. This use would allow this waste to be coined as a co-product rather than a by-product. Some of the uses of this co-product of the orange juice industry are the production of biopolymers, fertilisers, biofuels, or the extraction of pigments, essential oils, and some bioactive compounds for application in the chemical or cosmetic industry [11]. However, this last application still generates a by-product, which remains an environmental problem to be considered.

On the other hand, it should be taken into account that the co-product also has high water content (72.5%) [6], which makes it highly perishable, so some type of processing is necessary to stabilise it, while minimising the loss of nutritional and bioactive compounds. In this sense, freeze-drying could be an interesting technique, as it eliminates water by applying moderate temperatures, which is essential in the case of products with thermolabile compounds, such as bioactive compounds in fruits [12]. However, the dehydration of foods with a high sugar and acid content, such as fruits, leads to a product that quickly collapses and develops a sticky structure with high hygroscopicity [13]. To solve this problem, high molecular weight solutes can be added to the product to improve its stability and facilitate its storage and marketing [14]. In this study, gum Arabic (GA) and starch derivatised with octenyl succinate (OSA) were used for this purpose [12]. They also have been applied as thickeners, stabilisers, emulsifiers, and flavour encapsulants of confectionary and different beverages [15]. These biopolymers (Bp) have also been described as acting as encapsulating agents, helping to prevent the degradation of bioactive compounds. However, the functionality of the different biopolymers can affect the stability and the ease with which these bioactive compounds are released from the food matrix during digestion to be finally absorbed by the organism. Thus, bioaccessibility studies are critical to determining the remaining bioactive properties in food.

In this regard, this study focuses on the possible protective effect of GA and OSA at two different concentrations (0.25 and 0.45 g/g orange juice co-product dry solutes, ds), on the different bioactive compounds of the orange juice co-product before (OJC) and after freeze-drying (OJC-FD): vitamin C (VC), flavonoids hesperidin (HES) and narirutin (NAT), carotenoids (TC), total phenols (TP), and antioxidant capacity (AC), and especially from the point of view of their bioaccessibility, an aspect that has not been studied to date. The present study aimed to contribute to the interest of proposing the use of the freeze-dried co-product of the orange juice industry for use in human food, as an integrated zero-waste process, to ensure environmental protection and contribute to a sustainable and healthy diet. This would contribute to several of the United Nations Sustainable Development Goals (SDGs) to achieve a better and more sustainable future for all, specifically SDGs 1, 2, 3, and 12 [16].

## 2. Results and Discussion

The water content (x_w_) of the orange juice co-product and of the different freeze-dried orange juice co-product samples appears in Table 1. OJC showed a water content higher than x_w_ found in the literature for orange peel (72.5 g/100 g) [17], possibly because some pulp remained in the co-product used as raw material in this work after orange juice extraction. The freeze-drying process decreased (*p* < 0.05) the water content by around 95%, to 3.12–4.46 g/100 g, similar values to previous studies [18] and of the same order of those suggested by other authors for quality freeze-dried products [19].

### 2.1. Effect of the Presence of Biopolymers on the Bioactive Compounds Content and Antioxidant Capacity of Freeze-Dried Orange Juice Co-Product Samples

The effect of freeze-drying on TP, flavonoids, and TC appears in Table 1. OJC showed 117 ± 10 mg GAE/100 g co-product, in the range of the values obtained for orange peel (flavedo and albedo) in other studies (100–180 mg GAE/100 g) [7]. Phenolic compounds are essential components of fruit quality due to their contribution to flavour, colour, and functional properties. Among foods’ most important characteristics of polyphenols are their antioxidant properties. These antioxidant properties are one of the main reasons for their health effects, preventing diseases associated with oxidative stress, such as cancer, cardiovascular diseases, and neurodegenerative disorders [20]. Comparing TP content in whole oranges, the peel has more of these compounds than the pulp, as described by other authors [21]. Different authors have reported a similar observation in various citrus by-products [22]. Escobedo-Avellaneda et al. [7] stated that in the juice, there is an abundance of bound phenols, while in the pulp, albedo, and flavedo, the free phenols stand out. Freeze-drying led to an increase (*p* < 0.05) in the amount of TP, especially in the formulations with higher Bp. In this sense, TP increased by ≈4% in OJC-FD, ≈20% in Bp25, and ≈35% in Bp45, with respect to OJC (Equation (1)). These increases may be due to the greater ease of extraction associated with the high porosity of the freeze-dried co-products [12,21].

As regards flavonoids, they are an important class of polyphenols with diverse biological activities [23,24]. Its content is also higher in the peel than in the orange pulp. The dominant flavonoids in oranges are the glycosylated flavanones HES and NAT, both are primarily located in the peel [7]. In the Supplementary Materials, a representative chromatogram is shown in Figure S1. In general, freeze-dried orange juice co-products showed higher concentrations of HES and NAT than OJC (*p* < 0.05). In addition, incorporating biopolymers also resulted in a higher amount (*p* < 0.05) of flavonoids. Specifically, HES increased by ≈64% in OJC-FD, ≈100% in Bp25, and ≈140% in Bp45, and NAT by ≈22% in OJC-FD, ≈35% in Bp25 and ≈65% in Bp45, in relation to OJC (Equation (1)). This could be again attributed to an improved yield promoted by the freeze-drying process, which involved changes in the product porosity, thus facilitating the flavonoids extraction from the food matrix, as it has already been seen in previous studies [25]. 

**Figure 1:**
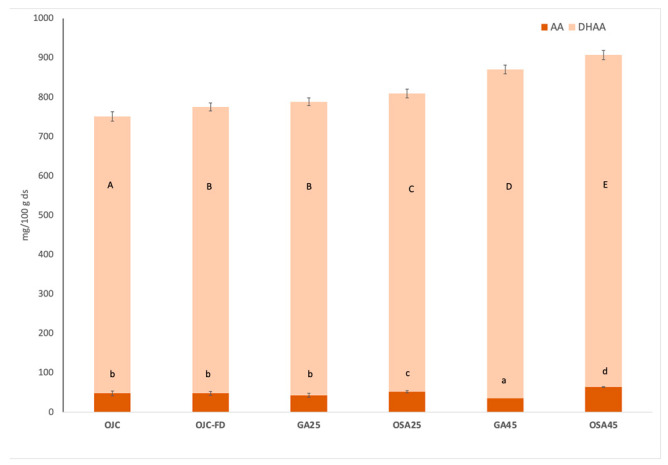
Vitamin C (sum of ascorbic acid (AA) and dehydroascorbic acid (DHAA)) of the orange juice co-product before (OJC) and after freeze-drying (OJC-FD), and those freeze-dried orange co-products formulated with gum Arabic and starch modified with octenyl succinic anhydride, at 25 and 45 g/100 g co-product dry solutes (GA25, GA45, OSA25, OSA45, respectively). Results are expressed as mg of bioactive/100 g of orange coproduct dry solutes (ds). Different letters indicate different homogeneous groups established by Tukey HSD ANOVA (*p* < 0.05) between samples: ^a–d^ for AA and ^A–E^ for DHAA.

Figure 1 shows the samples’ VC, ascorbic acid (AA), and dehydroascorbic acid (DHAA). Figure S2 illustrates an example of an obtained chromatogram (see Supplementary Materials). VC is found in two forms, AA (reduced form) and DHAA (oxidised form, also with vitamin action). In addition to preventing oxidative damage, vitamin C is also associated with preventing chronic degenerative illnesses, such as cardiovascular disease and cancer [26]. It should be noted that the majority form of VC in the orange co-product samples was DHAA. The last compound dominates in orange peels, contrary to what occurs in the pulp [24]. The VC obtained values for OJC (83 mg/100 g co-product) were lower than those published for orange peel by USDA [17] (136 mg/100 g) and higher than those reported in previous studies for orange pulp (58 mg/100 g) [12]. Other researchers [7] found that the orange peel contained greater VC values than the juice, and that the ratios of AA and DHAA varied between them. Thus, almost all of the VC in the juice was composed of AA, while DHAA made up the VC in the peel. The co-products with added biopolymers showed higher VC (*p* < 0.05). In previous studies, this vitamin has also been reported to have certain thermal stability [27]. In this case, VC also showed greater ease of extraction in the freeze-dried co-products, and an increase in DHAA was also observed (*p* < 0.05). Samples with higher biopolymers concentration increased ≈13% (GA45) and ≈20% (OSA45) VC content (*p* < 0.05) with respect to OJC (Equation (1)). Regarding the impact of the different Bp added on the stability of VC during freeze-drying, OSA seems to be more effective for the encapsulation of this vitamin. In this sense, GA is composed of polysaccharides and proteins, and according to other studies [28], DHAA reacts with proteins in low-water systems to produce L-Scorbamic acid and other degradation products. Silva-Spinoza et al. [12] also indicated better retention of VC in orange pulp freeze-dried powder when OSA was added to the formulation.

The TC content (Table 1) obtained for OJC (3.2 mg β-carotene/100 g co-product) was higher than the 0.3 mg/100 g value reported by Gardner et al. [29] for orange juice. Other studies have shown that orange flavedo has more carotenoid content than the edible fraction [7]. Several epidemiological studies positively correlate a higher dietary intake of carotenoids with a lower risk of cardiovascular diseases, macular degeneration and cancer [30]. TC carotenoids were degraded during the freeze-drying process, with general losses of 39% (Equation (1)). In this case, the addition of biopolymers does not seem to protect them. Other studies obtained similar carotenoid losses in the orange pulp during freeze-drying [12]. Due to the unsaturated nature of carotenoids, they are subject to changes mainly due to oxidation [31]. Fratianni et al. [32] evaluated the degradation of carotenoids in orange juice under different time/temperature conditions and concluded they were stable at temperatures of 60 to 70 °C. In this sense, the TC losses observed in this work may be more due to oxidation produced during sample processing, the effect of light, enzymes, and so forth, than to the freeze-drying temperature. 

**Figure 2:**
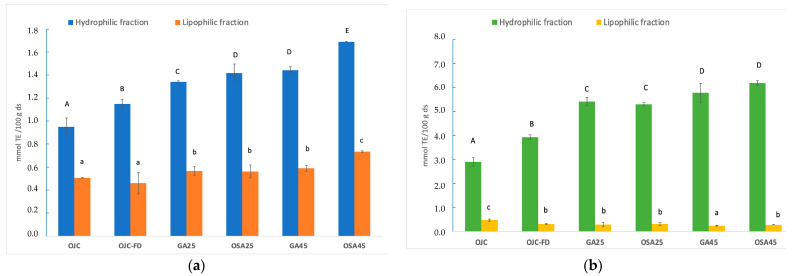
Antioxidant capacity measured by DPPH (**a**) and FRAP (**b**) of the hydrophilic and lipophilic fractions of orange co-product before (OJC) and after freeze-drying (OJC-FD), and those freeze-dried orange co-product samples formulated with gum Arabic and starch modified with octenyl succinic anhydride, at 25 and 45 g/100 g co-product dry solutes (GA25, GA45, OSA25, OSA45, respectively). Results are expressed as mmol Trolox Equivalent (TE)/100 g of orange coproduct dry solutes (ds). For each fraction, different letters indicate different homogeneous groups established by Tukey HSD ANOVA (*p* < 0.05) between samples: ^A–E^ for the hydrophilic fraction; ^a–c^ for the lipophilic fraction.

The composition and concentration of antioxidant compounds, such as vitamins, phenols, and carotenoids, determine a fruit’s AC. According to different studies, citrus AC is mainly attributed to its hydrophilic fraction, including VC, flavonoids, and some phenols [33]. The ability of many phenolic compounds and VC to donate hydrogen atoms from the hydroxyl groups in their ring structures is related to their reducing capacity [34]. Due to the different antioxidant mechanisms and the synergistic reactions between bioactive compounds, there is currently no single measurement method to assess the antioxidant capacity of a food. This makes it necessary to apply different assays to generate a profile of the antioxidant capacity of the compound or food [35]. However, the AC results can only be compared using the same method since the antioxidant mechanism studied differs. DPPH analysis examines the availability of a substance to reduce the DPPH˙ radical, while the FRAP assay is based on the capacity to reduce Fe^3+^ to Fe^2+^. Figure 2 shows the results of the AC analysed by the DPPH (Figure 2a) and FRAP (Figure 2b) methods for both the water-soluble fraction obtained from the phenolic extraction and the lipid-soluble part obtained from the carotenoid extraction. Some authors indicated that the orange albedo has higher AC than the pulp due to the peel’s elevated amount of flavonoids and other phenolic compounds [7]. Moreover, other studies have also reported the antioxidant effect of the dietary fibre present in citrus fruits, primarily located in the peel. In this regard, the antioxidant effect of pectin seems to be related to the enhancement of endogenous enzymes and free radical scavenging [36]. In this work, for both AC methods, it was observed that the antioxidant activity provided by the hydrophilic fraction was higher than the lipophilic one, as other studies corroborate for orange pulp [12].

The changes observed in TP, HES, NAT and VC during freeze-drying are reflected in the analysis of the antioxidant activity of the hydrophilic fraction, which increased (*p* < 0.05) in freeze-dried co-products compared to OJC. The protective effect of the biopolymers on the bioactive compounds can also be seen by increasing this property in the samples with the highest amount of Bp. OSA45 was the sample with the highest (*p* < 0.05) AC. Antioxidant activity found for OJC was higher than those reported by USDA [17] for orange juice (726 μm Trolox/100 g) due to the high concentration of bioactive compounds in the orange peel. In this sense, compared with other fruits recognised as rich sources of antioxidants, such as red grapes (1837 μm Trolox/100 g) [17], the antioxidant capacity of orange co-product can be considered high. Concerning the lipid-soluble fraction, all the freeze-dried samples showed a decrease in AC values compared to OJC when measured by the FRAP method (losses of 33–45%, Equation (1)). A similar trend was observed for the carotenoid compounds responsible for this activity (Table 1). 

To explain the relationships in the different compounds quantified in this study with the AC and the relationships among them, statistical correlation analyses were performed. Pearson’s correlation coefficients (r) are detailed in Table S1 (see Supplementary Materials). Except for ascorbic acid, all the hydrophilic bioactive compounds studied showed a strong positive significant Pearson’s correlation with each other and with AC (DPPH and FRAP) (*p* < 0.05). This could suggest that phenolics and VC, especially in its oxidised form DHAA, played a crucial role in the AC of orange co-products. Other authors have observed this behaviour in citric products [37,38]. In orange juice and pulp, it is widely accepted that AC is mainly related to VC and TP content [39,40]. Regarding the antioxidants analysed in the lipophilic fraction, CT was positively correlated with AC measured by the DPPH method (r = 0.9409; *p* < 0.05). Accordingly, orange carotenoids have demonstrated a scavenging capacity of the DPPH free radical [41], but no similar association was observed between carotenoids and FRAP [29].

### 2.2. In Vitro Bioaccessibility of Bioactive Compounds from Freeze-Dried Orange Juice Co-Product Samples with and without Biopolymers

The release of bioactive compounds in the intestinal digestion stage of the freeze-dried orange co-products appears in Table 1. Based on this content and the amount of bioactives present in the corresponding undigested samples, the bioaccessibility (Equation (3)) of these compounds in the different formulations of freeze-dried co-products was calculated (Table 2). This parameter is affected by bioactive substances’ modifications upon reaching the gastro-intestinal tract, influencing their absorption. Kamiloglu et al. [42] emphasised the importance of evaluating the availability of antioxidants after digestion due to evidence of poor bioavailability of certain antioxidants, which would, in turn, have a limited effect on health. A part of the bioactive substances is released from the food matrix in the upper part of the gastro-intestinal tract by direct solubilisation in the intestinal fluids under physiological conditions (37 °C, pH 1–7.5) and/or by the action of digestive enzymes so that the enzymatic hydrolysis of proteins, carbohydrates, and lipids promotes the release of the phytochemicals from the food matrix. These bioaccessible bioactive substances are partially absorbed through the mucosa of the small intestine. Another part of the ingested antioxidants (the non-bioaccessible amount) passes undissolved and unchanged through the upper intestine in association with dietary fibre. It reaches the colon, where it can be fermented by bacterial enzymes [43].

In general, bioactive compounds decreased compared to their concentration in the freeze-dried orange co-products before digestion (Table 1). To understand the TP decrease in the intestinal digest, it is necessary to consider that the alkaline pH of the intestinal phase causes phenolic compounds to undergo different chemical reactions, mainly oxidation and polymerisation, favouring the formation of other derived phenolic compounds (such as chalcones) that cannot be absorbed due to their low solubility and high molecular weight [33]. On the other hand, it has been described how interactions between phenolic compounds and other orange constituents (minerals, fibre) can favour the formation of complexes unable to cross the dialysis membrane [33]. Other studies also suggested that the reduction of phenolics after gastro-intestinal digestion is related to their sensitivity to slightly alkaline conditions (small intestine). It can promote the transformation of the structure of these compounds and influence their chemical properties and biological activity [44]. It should also be considered that orange peel contains a high amount of dietary fibre, which binds to phenols and prevents them from being absorbed through the walls of the intestine. The fibre-phenol complex passes into the large intestine, where they are degraded by the intestinal microbiota to simple phenolic acids that can be absorbed by the circulatory system or exert their beneficial effect by serving as a fermentation substrate for the intestinal microbiota. In this process, various compounds (acetate, propionate and butyrate, ammonia, gases, H_2_, CO_2_, amines, phenols), energy, and biomass are ultimately produced [45]. This mechanism makes possible a function essential for the intestinal ecosystem: the maintenance of the colonic microbiota and the enhancement of the immune system. The bioaccessibility of TP determined in the current study was lower than those found by Silva-Spinoza et al. [12] for freeze-dried orange pulp due to the different nature of phenols in the pulp and the peel. In this sense, some phenolic compounds could remain unchanged during digestion, and others could change their chemical structure due to pH changes. Particularly, citrus phenolic acids, such as ferulic acid, seem to increase after digestion, and others, such as vanilli and p-coumaric acids, decrease after digestion [46].

Notably, the compounds that were least bioaccessible were flavonoids HES and NAT. The reduction of flavonoids at the end of the digestion compared to non-digested samples has been observed for different fruits [47]. Specifically, studies on mandarin and orange peel showed that flavonoids were sensitive to pH conditions during in vitro gastro-intestinal digestion [8,48]. Thus, glycosylated flavonoids HES and NAT are more difficult to absorb than those with an aglycone structure, as the sugar part (glycoside) attached to the flavonoid skeleton must first be removed. In this sense, it has been described how hesperidin, quercetin, or catechin decrease during gastric digestion [43]. Most of the glycosylated flavonoids are resistant to acid hydrolysis in the stomach and reach the small intestine intact, where they are hydrolysed by the enzyme β-glucosidase. The remaining part, still undigested, passes to the colon to be digested by the microbiota to make assimilation possible [33].

On the other hand, incorporating biopolymers seems to improve the bioaccessibility of the studied biocompounds. The intestinal digests of the freeze-dried samples formulated with biopolymers showed higher amounts of TP, flavonoids and TC than OJC-FD digest (*p* < 0.05). These bioactive compound release may exert health benefits at the intestinal level. However, VC was not detected in any intestinal digest, showing the lability of this vitamin susceptible to factors such as pH, enzymes, light, and temperature, to which samples are subjected during simulated in vitro gastro-intestinal digestion. Moreover, as already mentioned, in the orange juice co-products VC was mostly found in its oxidised form (DHAA), it is much more susceptible to transformation to oxidised forms (2,3-diketogulonic acid) without vitamin action. The VC oxidation in the gastro-intestinal tract is due to its action as an electron donor maintaining ions in the reduced state and regenerating the active form of other dietary constituents.

## 3. Materials and Methods

### 3.1. Materials

As raw material for this study, orange (*Citrus sinensis* (L.), var. Navelina) juice waste (Speed Up, Zumex, Valencia, Spain) was provided by the cafeteria of the Facultat de Belles Arts Sant Carles at the Universidad Politécnica de València (Spain), in October 2021. The biopolymers used as encapsulating agents were GA (Scharlab, Sentmenat, Spain) and OSA (Roquette, Benifaió, Spain). Chemicals and enzymes used for the in vitro digestion, the gallic acid used for analysing the total phenolic compounds, the DL-dithiothreitol reagent for the vitamin C analysis, and the Trolox used for the antioxidant activity assays were obtained from Sigma-Aldrich (Saint Louis, MO, USA). The β-carotene used for total carotenoids for analysis was obtained from Dr. Ehrenstorfer (Augsburg, Germany). For main flavonoid quantification, hesperidin and narirutin were used (TCI Europe N.V., Paris, France). L(+) ascorbic acid standard used for the vitamin C analysis and dimethylsulfoxide grade HPLC for flavonoid extraction were obtained from Scharlau SL (Sentmenat, Spain). All the solvents used for the extraction of the bioactive compounds were obtained from VRW International (Barcelona, Spain). 

### 3.2. Preparation of Powdered Co-Product Samples

After cleaning with running water, the orange juice co-product was sliced into four portions, and water was added for easy handling (100:37.8, *w*/*w*). Then it was emulsified for five minutes (Robot coupe blixer, Burgundy, France) to obtain a homogeneous blend. A part of the orange juice co-product was formulated with GA or OSA at two concentrations of biopolymers: 0.25 or 0.45 g Bp/g ds. For that, different solutions of each Bp in distilled water were made 24 h earlier. These formulations were prepared in accordance with Martínez-Navarrete et al. [49]. The mixtures were placed on aluminum trays (25 cm diameter, thickness 1 cm) and frozen at −45 °C (LGT 2325, Liebherr, Kirchdorf, Germany) for 48 h for subsequent freeze-drying. The frozen samples were freeze-dried (Telstar Lyoquest-55, Terrassa, Spain) at a pressure of 0.050 mbar, −50 °C in the condenser, and 50 °C on the shelves of the chamber for 21 h. The time was selected based on previous experiments, trying to obtain products with a water content of under 5%. The freeze-dried samples were ground (Thermomix TM 21, Vorwerk, Valencia, Spain), and the powder obtained was sieved (200 μm mesh, AMP0.40, CISA, Barcelona, Spain) and stored in zip bags under refrigeration conditions (4 °C) until analysis. This resulted in five formulations: OJC-FD, GA25 (0.25 g GA/g ds), GA45 (with 0.45 g GA/g ds), OSA25 (with 0.25 g OSA/g ds), and OSA45 (with 0.45 g OSA/g ds). 

### 3.3. In Vitro Gastro-Intestinal Digestion

The in vitro digestion protocol developed by Miller et al. [50], adapted to include the oral step [51], was applied to simulate the gastro-intestinal digestion of samples. All the digestions were performed in triplicate in a temperature-controlled chamber (Nüve, test cabinet TK 120, Ankara, Turkey) to ensure that the samples were at 37 °C and under constant agitation (Rotabit, J.P. SELECTA, Logroño, Spain) at 120 oscillations per minute (opm). Briefly, the digestion consisted of 3 sequential phases: the oral phase, 12 g of the corresponding freeze-dried samples + 120 mL water, were mixed with 250 μL of an α-amylase/CaCl_2_ solution (130 mg α-amylase/100 mL CaCl_2_ 1 mM, pH 7) per gram of solid. Samples were incubated at 37 °C for 10 min at 120 opm. In the gastric phase, the pH of samples (100 mL of oral digest) was adjusted to 2.0 with HCl 2 M. After that, 0.1 g of pepsin of porcine origin (40,000 units) was added. The mixture was incubated at 37 °C and 120 opm for 2 h. Finally, a dialysis membrane (14,000 D pore size) filled with 25 mL of 0.5 N NaHCO_3_ was used to simulate intestinal digestion. 20 mL of sample from the gastric step was dialysed under agitation at 37 °C. Once the sample reached pH 5, a volume of 5 mL of a mixture of pancreatin (4 g/L) and bile extract (25 g/L) in 0.1 N NaHCO_3_ was added, and incubation was continued for 2 h until pH 7.5 was reached. After the intestinal step, two fractions were collected: the external and internal parts of the dialysis membrane. The external content of the dialysis tube was considered to be the part of the digest that reached the colon, while the internal dialysis membrane contained the compounds capable of crossing the membrane, which was considered the bioaccessible fraction. The internal intestinal digest was collected and stored in sterile vessels at −45 °C.

### 3.4. Analytical Determinations

All analyses were performed in triplicate on the five freeze-dried formulations before and after the in vitro digestion. OJC was also characterised in the same parameters to be taken as control. The analyses were carried out as described below for every sample. In the case of the digests, they were previously centrifuged at 11,515× *g* at 10 °C for 10 min (GYROZEN Co., 1236R, GYROZEN, Daejeon, Korea) and the supernatant was filtered by a 45 µm nylon filter before each analysis. For comparative purposes, results were expressed per 100 g orange juice co-product ds [12]. 

Also, the changes in bioactive compounds caused by the freeze-drying process were calculated for freeze-dried orange co-product samples in reference to the OJC bioactive content (Equation (1)).

(1) $$\mathrm{Changes}\;\mathrm{of}\;\mathrm{bioactive}\;\mathrm{compounds}\;\left(\%\right)=\frac{\left[\mathrm{BcOJC}-{\mathrm{BcFD}}_{x}\right]\;}{\left[\mathrm{BcOJC}\right]\;}\times 100$$

where BcOJC is the bioactive compound content in the OJC sample (mg/100 g ds), and BcFD_x_ is the bioactive compound content in each freeze-dried orange co-product (mg/100 g ds). 

#### 3.4.1. Water Content

The water content (x_w_, g water/100 g product) of the OJC sample before being freeze-dried was determined by drying in a vacuum oven (Selecta^®^, Vaciotem-T, J.P. Selecta S.A., Barcelona, Spain) at 60 ± 1 °C under *p* < 100 mm Hg until constant weight (XS204 DeltaRange^®^, Mettler Toledo, Switzerland) [52]. For the freeze-dried samples, the x_w_ was determined by using an automatic Karl Fisher titrator (Mettler Toledo, Compact Coulometric Titrator C10S, Worthington, OH, USA). 

#### 3.4.2. Total Phenolic Compounds

TP was estimated using the spectrophotometric Folin–Ciocalteu assay with some modifications [12]. For TP extraction, samples were mixed with 9 mL of methanol: water (70:30 *v*/*v*) solution at 200 rpm (magnetic multi-stirrer MS-51M, Jeio Tech, Seoul, Korea), in darkness and at room temperature for 30 min. The homogenates were centrifuged at 11,515× *g* at 4 °C for 10 min (GYROZEN 1236R, Daejeon, Korea), and the upper layer was collected for analysis. Gallic acid was used for the calibration curve, and the data were expressed as mg of gallic acid equivalent (GAE)/100 g ds. 

#### 3.4.3. Major Flavonoids: Hesperidin and Narirutin

The analysis of HES and NAT were carried out by using ultra-high-performance liquid chromatography (UHPLC) equipment connected to a DAD detector (Jasco equipment, Italy) and a Synergi 4 μm Hydro-RP column (Phenomenex, Madrid, Spain). Methanol and HPLC-grade water were used as the mobile phase. The flow rate was 1 mL/min, and the injection volume was 10 μL. Flavonoids were extracted by mixing the samples with 1 mL of double-distilled water and 2 mL of dimethylsulfoxide under magnetic agitation (200 rpm) for 10 min (MS-51M, Jeio Tech, Seoul, Korea) [24]. Subsequently, the solution was centrifuged at 2031× *g* (GYROZEN 123 GR, Daejeon, Korea) for 10 min at 4 °C. The supernatant was filtered through a 0.45 μm membrane filter for injection into the UHPLC. The flavonoid identification was performed at wavelength 284 nm, and proper patterns were used for quantification.

#### 3.4.4. Vitamin C

The determination of VC consisted of the reduction of dehydroascorbic acid to ascorbic acid (AA) using DL-dithiothreitol as a reducing agent [24] and a UHPLC determination [24]. The conditions were: Kromaphase100-C18, 5 mm (4.6 × 250 mm) column (Scharlau SL, Sentmenat, Spain); mobile phase 0.1% oxalic acid, volume injected 10 μL, flow rate 1 mL/min, detection at 243 nm (detector UV-visible MD-1510, Jasco, Cremella, Italy) at 25 °C. VC was identified by its retention time and quantified by integrating the areas of the peaks obtained from the chromatograms using AA as standard. 

#### 3.4.5. Total Carotenoids

The TC extraction was performed under the same conditions as for TP but using as extraction solvent a hexane: acetone: ethanol (50:25:25, *v/v*) mixture [12]. For quantification spectrophotometry method was used [52], absorbance was measured at 446 nm (spectrophotometer V-1200 VWR, VWR, Radnor, PA, USA), and β-carotene standard was used for the calibration curve.

#### 3.4.6. Antioxidant Activity 

The AC was determined in the extracts obtained for the determination of TP and TC to consider the contribution of the hydro-soluble and lipid-soluble fractions. Two complementary assays, FRAP and DPPH, were used, and results were converted to mmol Trolox equivalent (TE)/100 g ds. 

The FRAP method (Ferric-reducing antioxidant power) was performed according to previous studies [12], and the absorbance was measured at 593 nm (V-1200 VWR, VWR, Radnor, PA, USA).

For the DPPH scavenging capacity assay [12], the degree of discolouration of the DPPH radical by the action of antioxidants was measured at 515 nm (V-1200 VWR, VWR, Radnor, PA, USA) at the initial time (A_control_) and after 15 min of reaction (A_sample_). The percentage of DPPH was calculated following Equation (2).

(2) $$\%\mathrm{DPPH}=\frac{\left(A_{\mathrm{control}}-A_{\mathrm{sample}}\right)}{A_{\mathrm{control}}}\times 100$$

#### 3.4.7. In Vitro Bioaccessibility

Bioaccessibility, defined as the portion of bioactive compounds released from the food matrix into the gastro-intestinal tract and thus become available for intestinal absorption, was determined in the bioaccessible fraction. For that, in each digestion, an aliquot of the dialysed intestinal digest was collected at the end, and the in vitro bioaccessibility of the different bioactive compounds was calculated following Equation (3) [12].

(3) $$\mathrm{Bioaccesibility}\left(\%\right)=\frac{\left[C_{d}\right]\;}{\left[C_{x}\right]\;}\times 100$$

where, C_d_ is the concentration of the bioactive compounds found in the bioaccessible fraction after the in vitro digestion, whereas C_x_ is the concentration in the corresponding non-digested freeze-dried orange co-product sample.

### 3.5. Statistical Analysis

All data are presented as means ± standard deviation for three replicates of each sample. Statgraphics Centurion XVII software (Statgraphics Technologies, Inc., The Plains, Virginia) was used to perform a univariate analysis of variance (ANOVA) with Tukey’s HSD test. *p* < 0.05 was used to determine whether the differences were significant.

## 4. Conclusions

The freeze-dried orange juice co-product showed high content of vitamin C (especially in the form of dehydroascorbic acid), total phenols, flavonoids (hesperidin and narirutin) and carotenoids (β-carotene), with antioxidant properties. In this sense, this co-product has a potential functional value that could have interesting applications in food technology.

The valorisation of this co-product of the orange juice industry for human food would promote environmental protection, economic development and, at the same time, contribute to a sustainable and healthy diet. Adding gum Arabic and modified starch to the orange juice co-product improved the stability of hydrophilic bioactive compounds (total phenols, flavonoids, and vitamin C) during freeze-drying. This preservative effect was higher for higher biopolymer concentrations (0.45 g/g orange coproduct dry solutes). However, no protective effect on carotenoid compounds was observed. This trend was reflected in the antioxidant activity of the different samples. 

The in vitro simulation of digestion showed that the incorporation of biopolymers improved the bioaccessibility of the bioactive compounds studied. The addition of gum Arabic and modified starch is therefore recommended, as their incorporation seems to prevent, to some extent, the degradation of the studied bioactive compounds and their antioxidant properties during freeze-drying, and improves their release during digestion to be absorbed by the organism. From this point of view, it would be interesting to continue this research by performing in vivo studies to confirm the results obtained in vitro.

## Figures and Tables

**Table 1:** Values (mean ± standard deviation) of water content (x_w_, g water/100 g sample) and bioactive compounds of the orange co-product before (OJC) and after freeze-drying (OJC-FD), and those of freeze-dried orange co-products formulated with gum Arabic and starch modified with octenyl succinic anhydride, at 0.25 and 0.45 g/g orange co-product dry solutes (GA25, GA45, OSA25, OSA45, respectively) The effect of the in vitro digestion on the bioactive compounds is also shown.

Sample		x_w_	TP ^1^	Hes ^2^	Nat ^3^	TC ^4^
OJC		88.99 ± 0.05 ^b^	986 ± 92 ^a^	2681 ± 37 ^a^	288 ± 20 ^a^	28 ± 4 ^b^
OJC-FD	BD	4.41 ± 0.07 ^a^	1024 ± 16 ^b^	4403 ± 50 ^b^	351 ± 7 ^b^	17.8 ± 0.4 ^a^
	AD		157 ± 7 ^A^	16 ± 0 ^A^	7.9 ± 0.1 ^A^	0.9 ± 0.1 ^A^
GA25	BD	3.42 ± 0.08 ^a^	1187 ± 10 ^c^	5325 ± 31 ^c^	370 ± 2 ^b^	16.7 ± 0.6 ^a^
	AD		238 ± 7 ^B^	20.7 ± 0.1 ^B^	12.4 ± 0.1 ^B^	0.9 ± 0.9 ^A^
OSA25	BD	3.46 ± 0.07 ^a^	1145 ± 7 ^c^	5548 ± 15 ^c^	405 ± 7 ^c^	14.0 ± 0.2 ^a^
	AD		238 ± 6 ^B^	22.9 ± 0.1 ^C^	13.1 ± 0.1 ^B^	1.1 ± 0.6 ^B^
GA45	BD	3.32 ± 0.07 ^a^	1369 ± 78 ^d^	6533 ± 46 ^d^	520 ± 27 ^d^	14.3 ± 0.3 ^a^
	AD		323 ± 2 ^C^	26.6 ± 0.1 ^D^	16.3 ± 0.1 ^C^	1.27 ± 0.7 ^C^
OSA45	BD	3.53 ± 0.05 ^a^	1321 ± 87 ^d^	6318 ± 42 ^d^	458 ± 15 ^d^	14.9 ± 0.3 ^a^
	AD		285 ± 3 ^D^	28.9 ± 0.1 ^E^	17.2 ± 0.1 ^D^	1.67 ± 0.6 ^D^

BD: Before digestion. AD: After digestion. ^1^ Total phenolic compound, referred to gallic acid equivalent; ^2^ Hesperidin; ^3^ Narirutin; ^4^ Total Carotenoids, referred to β-carotene. Results are expressed as mg of bioactive/100 g of orange coproduct dry solutes. Different letters in superscripts per column indicate different homogeneous groups established by Tukey HSD ANOVA (*p* < 0.05) between samples: ^a–d^ for non-digested samples; ^A–D^ for digested samples.

**Table 2:** Bioaccessibility of bioactive compounds (%, Equation (3)) of the freeze-dried orange juice co-products formulated without (OJC-FD) and with gum Arabic and starch modified with octenyl succinic anhydride, at 0.25 and 0.45 g/g co-product dry solutes (GA25, GA45, OSA25, OSA45, respectively).

Sample	OJC-FD	GA25	OSA25	GA45	OSA45
TP	15.29 ^a^	20.09 ^b^	20.83 ^b^	23.61 ^c^	21.62 ^c^
HES	0.36 ^a^	0.39 ^b^	0.41 ^b^	0.41 ^b^	0.46 ^c^
NAT	2.26 ^a^	3.33 ^b^	3.24 ^b^	2.94 ^b^	3.77 ^c^
TC	5.00 ^a^	5.42 ^a^	7.79 ^b^	8.89 ^b^	11.16 ^c^

Different letters in superscripts in the same row indicate significant differences established by the Tukey HSD ANOVA (*p* < 0.05). TP: total phenolic compounds; HES: hesperidin; NAT: narirutin; TC: total carotenoids.

## Data Availability

Not applicable.

## Supplementary Materials

The following supporting information can be downloaded at: https://www.mdpi.com/article/10.3390/molecules28020810/s1, Figure S1: Representative HPLC chromatogram of flavonoids narirutin and hesperidin, Figure S2: Representative HPLC chromatogram of vitamin C, Table S1: Pearson’s correlation coefficients between vitamin C (VC), ascorbic acid (AA), dehydroascorbic acid (DHAA), hesperidin (HES), narirutin (NAT), total phenols (TP), and antioxidant activity (AC) of the orange co-products hydrophilic fraction measured by DPPH and FRAP methods.
