# Supplementary material for: In Vitro Bioaccessibility of Bioactive Compounds of Freeze-Dried Orange Juice Co-Product Formulated with Gum Arabic and Modified Starch
Source: Molecules. 2023 Jan 13;28(2):810. doi: 10.3390/molecules28020810 (PMC9862788; doi:10.3390/molecules28020810)
Supplement: Supplementary file 1 [file molecules-28-00810-s001.zip › molecules-2157508-supplementary.pdf]

Supplementary Material

In Vitro Bioaccessibility of Bioactive Compounds of Freeze-Dried Orange Juice Co-Product Formulated with Gum Arabic and Modified Starch

Eva García-Martínez, María del Mar Camacho * and Nuria Martínez-Navarrete

Food Technology Department, Food Investigation and Innovation Group, Universitat Politècnica de València, Camino de Vera s/n, 46022 Valencia, Spain; evgarmar@tal.upv.es (E.G.-M.); nmartin@tal.upv.es (N.M.-N.)

* Correspondence: mdmcamvi@tal.upv.es

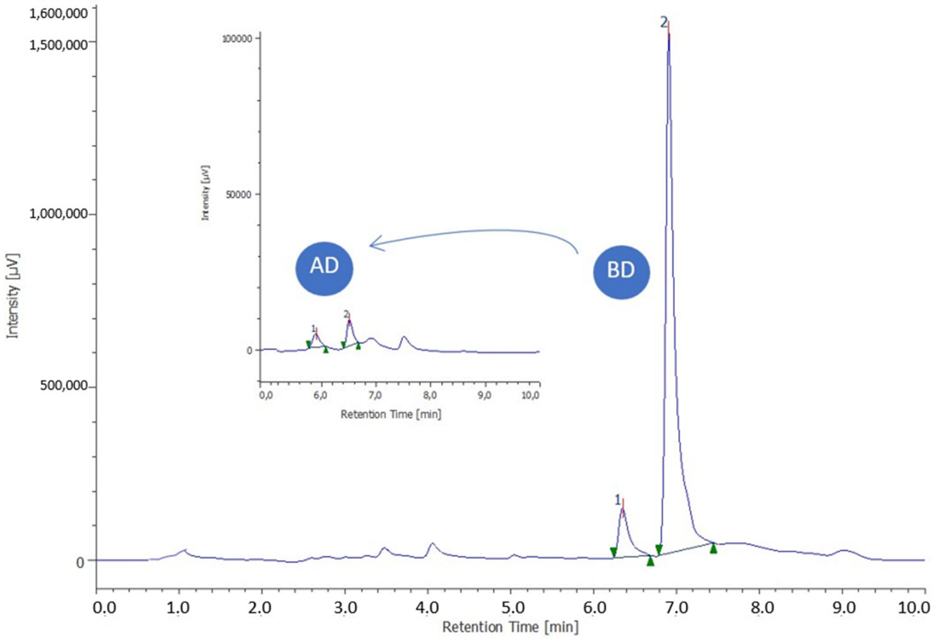

Figure S1. Representative HPLC chromatogram of flavonoids narirutin (1) and hesperidin (2). AD: after digestion; BD: Before digestion.

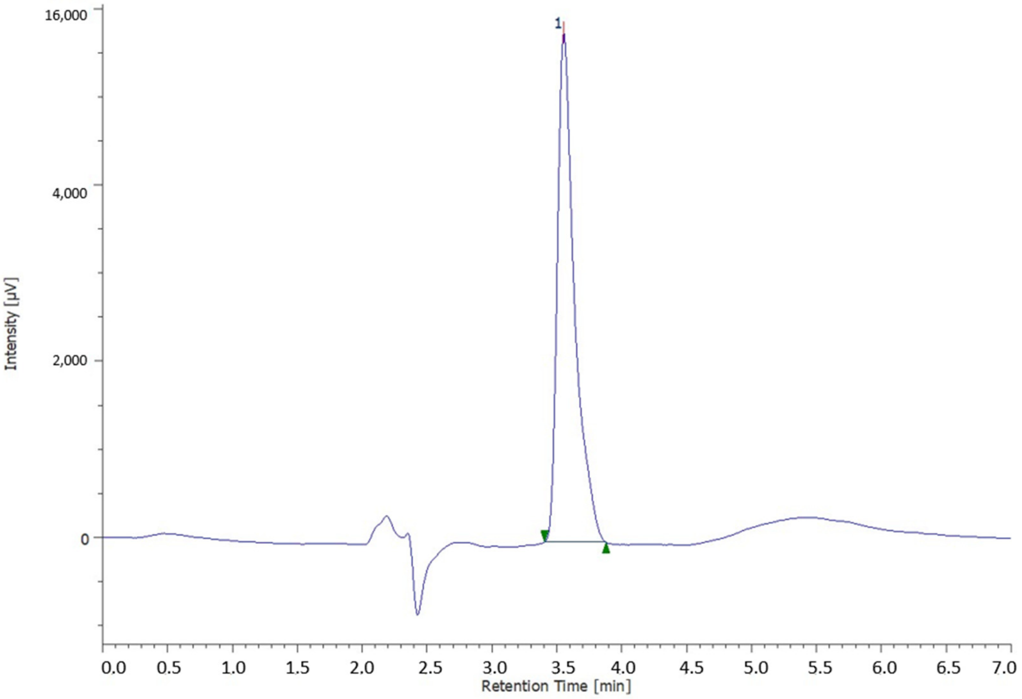

Figure S2. Representative HPLC chromatogram of vitamin C.

Table S1. Pearson's correlation coefficients between vitamin C (VC), ascorbic acid (AA), dehydroascorbic acid (DHAA), hesperidin (HES), narirutin (NAT), total phenols (TP), and antioxidant activity (AC) of the orange co-products hydrophilic fraction measured by DPPH and FRAP methods.

	**AC (FRAP)**	**VC**	**AA**	**DHAA**	**HES**	**NAT**	**TP**
AC (DPPH)	0.9634*	0.9453*	0.3826	0.8846*	0.9261*	0.8182*	0.8678*
AC (FRAP)		0.8661*	0.1454	0.8590*	0.9723*	0.8570*	0.9135*
VC			0.4113	0.9595*	0.8505*	0.8423*	0.8833*
AA				0.1485	0.0304	−0.1063	−0.0381
DHAA					0.8852*	0.9439*	0.9575*
HES						0.9317*	0.9208*
NAT							0.9472*

*—correlation is significant at $p \leq 0.05$ level.
